# Lung metastasis from renal cell carcinoma 16 years after nephrectomy: A case report and review of the literature

**DOI:** 10.1002/ccr3.5033

**Published:** 2021-11-06

**Authors:** Moushami Singh, Vinayak Aryal, Ashis Man Singh Dangol, Karun Neupane, Banita Gurung, Suniti Shrestha, Sampurna Man Tuladhar, Swechha Maskey, Hari Prasad Dhakal

**Affiliations:** ^1^ Department of Pathology and Laboratory Medicine Nepal Cancer Hospital and Research Center Lalitpur Nepal; ^2^ Department of Emergency Medicine Buddha MAI Center Buddhabari Teku Nepal; ^3^ Department of Internal Medicine Jacobi Medical Center Bronx New York USA; ^4^ Department of Cardiac and Thoracic Surgery Nepal Cancer Hospital and Research Center Lalitpur Nepal

**Keywords:** nephrectomy, pulmonary metastasis, pulmonary nodule, renal cell carcinoma

## Abstract

Renal cell carcinoma can have lung metastasis even after a long interval of radical nephrectomy (16 years after nephrectomy in our case). If any pulmonary nodule is diagnosed with a history of RCC, pulmonary metastasis of RCC should be suspected and should be appropriately treated.

## Graphical abstract

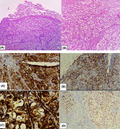

## 1 INTRODUCTION

The recurrence rate of renal cell carcinoma in the lungs in the first five years after nephrectomy is 93%. The occurrence of RCC after 16 years of nephrectomy as pulmonary metastasis is rare and needs to be considered in a patient presenting with pulmonary nodules even after a long interval since nephrectomy. Renal cell carcinoma (RCC) accounts for 3% of all adult cancers and 85% of all kidney tumors, making it the most common renal malignancy.^1^ With an incidence rate of 14.5 per 100,000, RCC is the seventh most common solid cancer and comprises 3%–5% of all adult malignancies.^1^, ^2^ It has a high recurrence rate of 93% in the first five years after nephrectomy.^3^ Lungs are the most common site for distant metastasis (50%–60%). The median time to recurrence is 15–18 months after nephrectomy. Eighty‐five percent of the relapse cases occur within 3 years of nephrectomy.^4^

Here we report a rare case of RCC that presented with lung metastasis 16 years after nephrectomy. Based on our literature review, we found only ten reported cases with intervals more prolonged than in our case between nephrectomy for RCC and lung metastasis: 17 years,^5^ 19 years,^6^ six cases with 20–28 years intervals,^7^ 31 years,^8^ and 37 years.^9^

## 2 CASE REPORT

**FIGURE 1:**
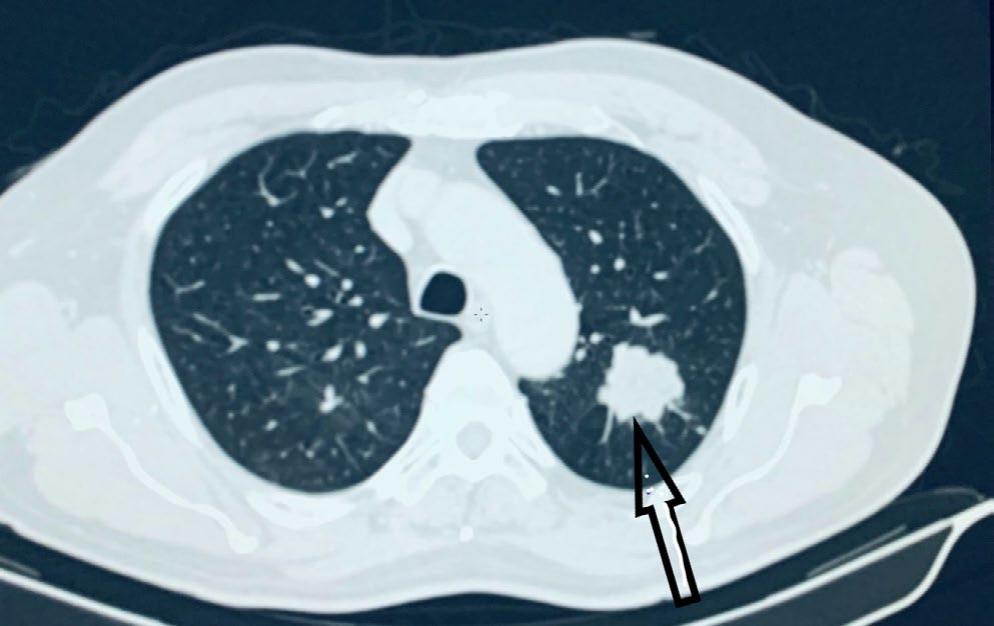
CT Scan of the chest shows a minimally enhancing lesion measuring 3.3 × 3.2 cm with lobulated margins(as indicated by an arrow)

A 70‐year‐old male presented with mild throat and chest discomfort for four months. A chest x‐ray was performed, which demonstrated a left upper lobe opacity. On further imaging, contrast‐enhanced computed tomography (CECT) chest demonstrated a heterogeneously enhancing oval soft tissue density lesion (3 × 3 × 2.9 cm) with a lobulated outline and slight speculation at places involving the apicoposterior segment of the upper lobe of the left lung (Figure 1). Bronchoscopy demonstrated normal findings. He had a history of renal cell carcinoma for which he underwent a right nephrectomy 16 years ago, vertebral fusion surgery 24 years ago, and cholecystectomy 17 years ago. He did not have a history of smoking. The patient was then re‐evaluated at our center with a CECT chest and abdomen, which showed a 3 × 3 × 3.2 cm lesion in the left upper lobe of the lung without enlarged mediastinal lymph nodes. There was no visible recurrence in the right kidney bed.

**FIGURE 2:**
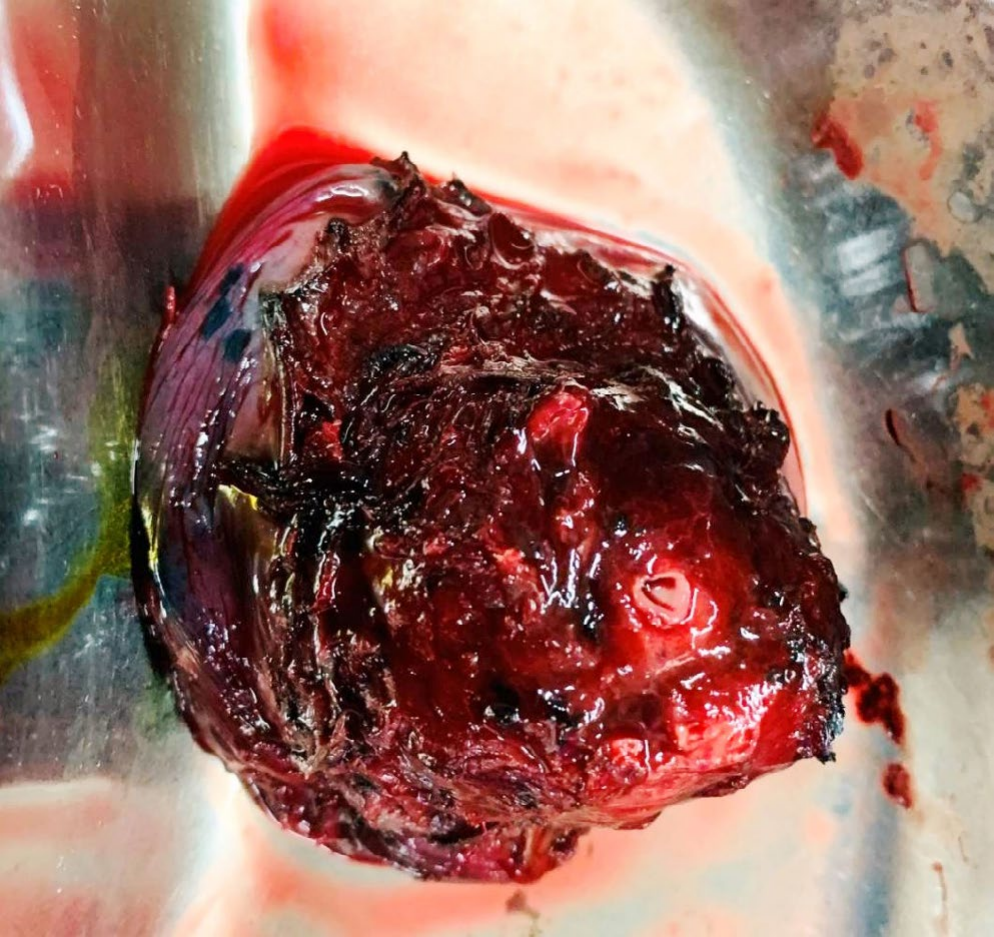
Surgical specimen of the resected lung mass

**FIGURE 3:**
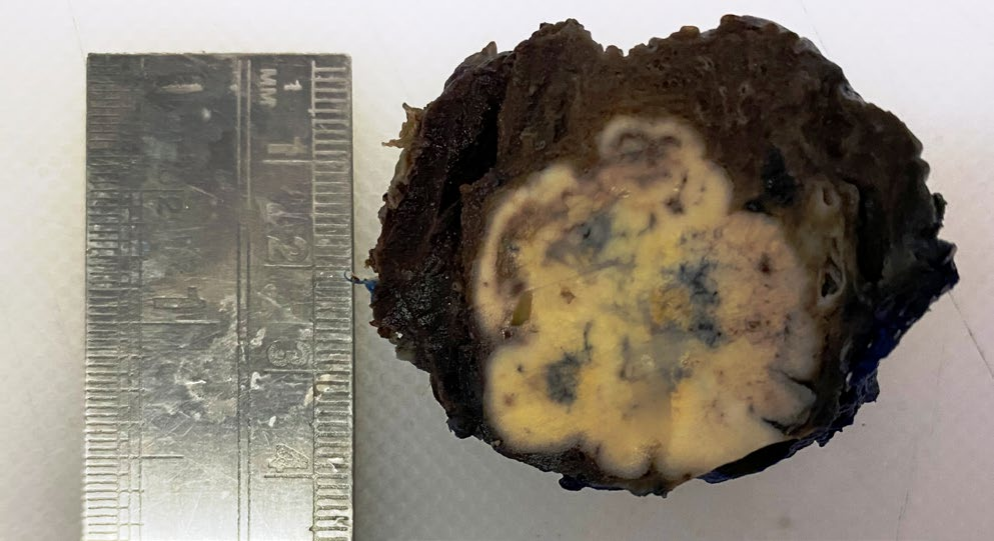
Gross specimen: On the cut section, the tumor is circumscribed, nodular, and yellowish in appearance

**FIGURE 4:**
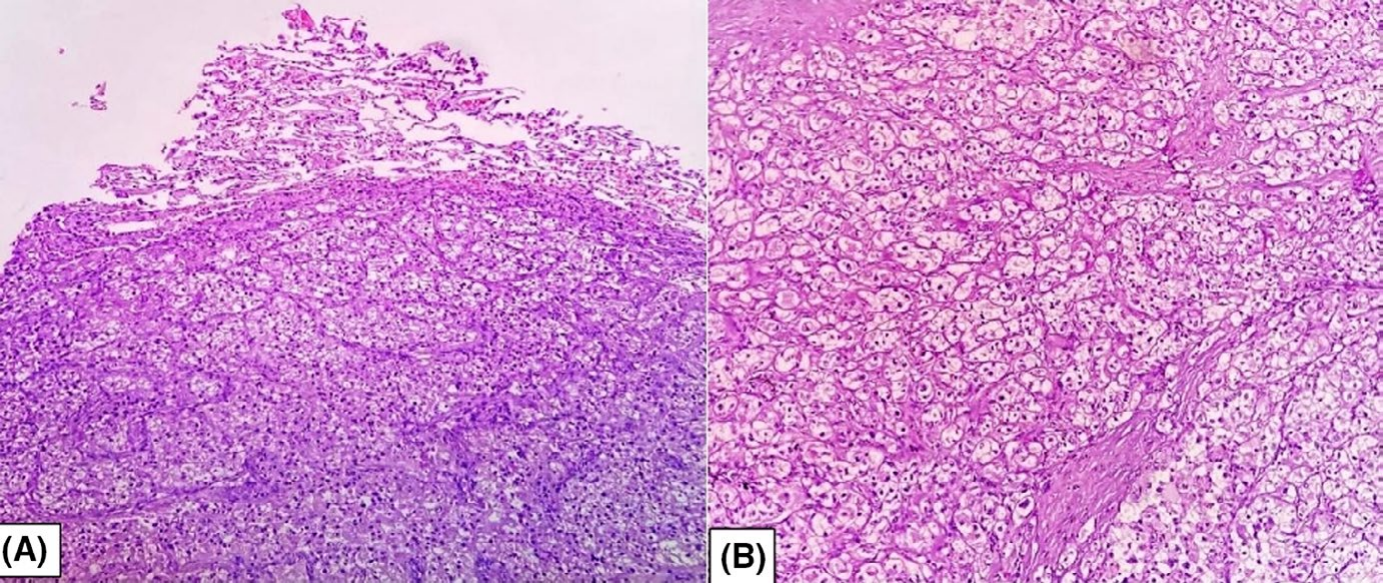
(A) 10× magnification photomicrograph showing clear cell renal cell carcinoma (ccRCC) in lung. (B) 40× photomicrograph showing tumor cells arranged in nests, have a polygonal shape with clear cytoplasm, and separated by thin‐walled blood vessels

**FIGURE 5:**
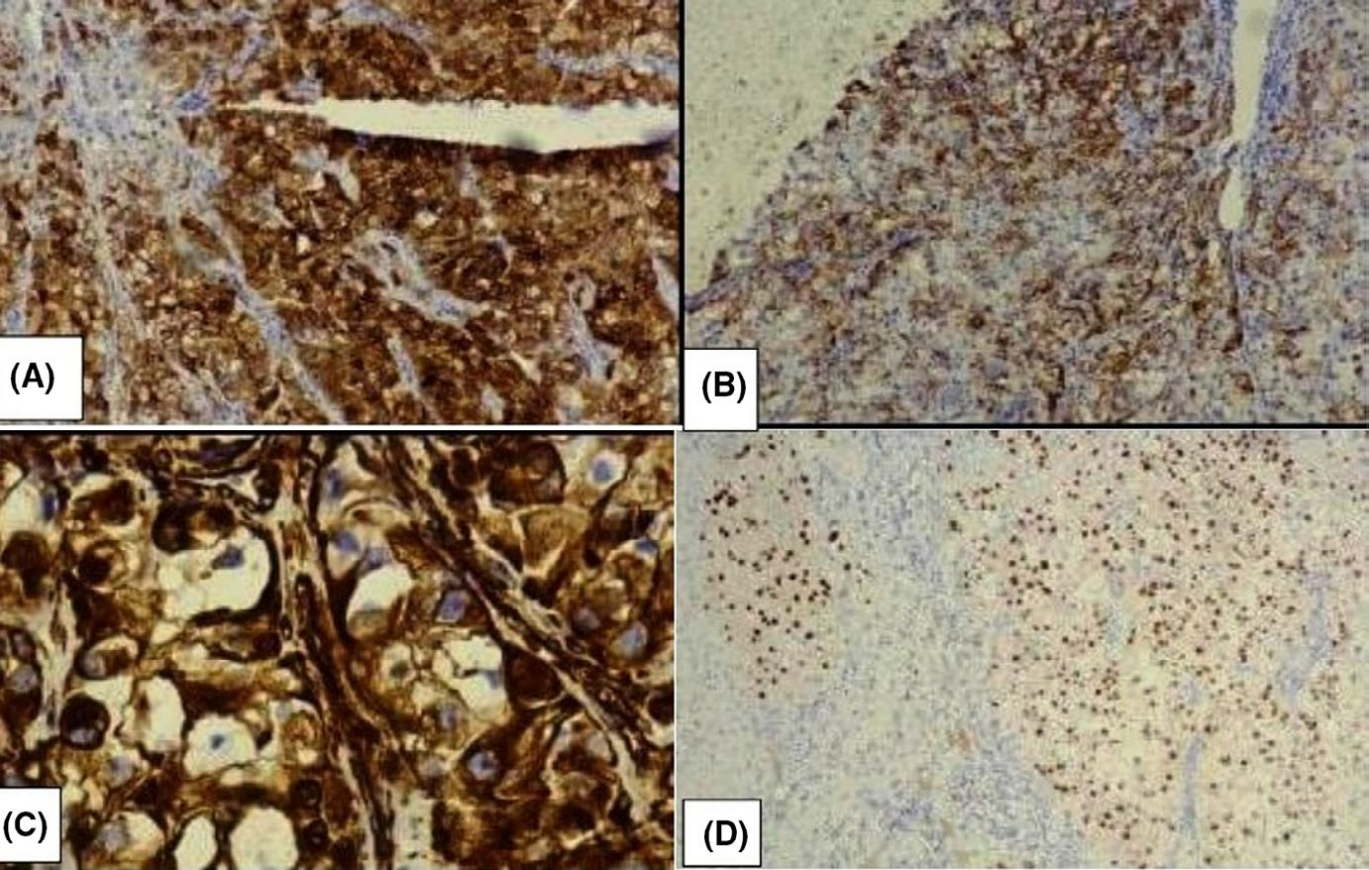
Tumor cells show positivity for (A) CD10, (B) pancytokeratin (CK AE1/AE3), (C) vimentin, and (D) PAX 8.

CT‐guided tru‐cut biopsy from the upper lobe of the left lung lesion was diagnosed as clear cell tumor favoring clear cell carcinoma. Later after a month, wedge resection of the left upper lobe of lung was done (Figure 2). Grossly, circumscribed tumor of maximum dimension of 3.5 cm was noted (Figure 3). Histological sections showed tumor cells arranged in solid sheets and nests. These tumor cells were pleomorphic, polygonal in shape with the delineated cell membrane, central nuclei with vesicular chromatin and abundant clear to eosinophilic cytoplasm. Some of the cells showed prominent eosinophilic nucleoli (Figure 4A,B). Immunohistochemistry was done to confirm the diagnosis further. On immunohistochemistry, tumor cells were positive for cytokeratin (CK AE1/AE3), paired box gene (PAX8), cluster of differentiation 10 (CD10), vimentin, and weakly focal positive PAX2 and negative for CK7, CK20, thyroid transcription factor 1 (TTF1) and P40 (Figure 5). The histomorphological features along with immunohistochemical analysis favored the diagnosis of metastatic clear cell renal cell carcinoma.

The postoperative period was uneventful. The patient is currently under chemotherapy (sunitinib 2 weekly) and has completed the ninth cycle to date. Evaluation with CT scan of the chest and abdomen on the last follow‐up did not show any recurrence. The patient is stable without any new symptoms at 11 months of follow‐up

## 3 DISCUSSION

RCC is the sixth most common malignant tumor in males and the tenth most common malignant tumor in females.^10^ RCC recurs after more than ten years of nephrectomy in 10% of the cases.^11^ The most common sites of metastasis include lungs, bones, lymph nodes, adrenal glands, liver, brain, contralateral kidney, and pancreas.^5^ Metastatic clear cell RCC has a poor prognosis with a median overall survival of 12 months only which has improved in the last decade.^12^ Pulmonary metastasis from RCC after a long interval is rare. This is the first case of lung metastasis from renal cell carcinoma encountered in our institution. To the best of our knowledge, this is the first case of lung metastasis secondary to clear cell renal cell carcinoma reported from Nepal to date.

Shiono et al^7^ reported a case with repeated metastasectomy for lung metastasis 16, 24, and 25 years after RCC treatment. Shiono et al^7^ also reported a mini‐review of 5 cases of RCC with lung metastasis after 20–28 years of resection of primary neoplasm throughout 1973–1998. Watanabe et al^8^ reported pulmonary metastasectomy after 10 years of nephrectomy. Left upper lobectomy was performed in an 82‐year‐old patient who was consequently disease‐free at 9 months follow‐up. Tamburrini et al^9^ reported lung metastasis after 37 years of RCC resection. The patient had a central hilar mass suspected as primary lung cancer but histological examination revealed metastatic RCC. Left pneumonectomy was performed in this patient. The patient was recurrence‐free at 3 years follow‐up. Bradham et al. reported lung metastasis in a patient 25 years after nephrectomy for RCC.^13^

Disease recurrence likelihood for RCC depends on several known and unknown factors including host and tumor factors.^5^ Prognostic indicators for RCC include complete resection of all metastasis, low number of metastasis, long disease‐free interval between primary tumor diagnosis and lung lesions, lymph node involvement, and size of pulmonary nodule.^9^ Host immune response has been hypothesized to play a role in determining the time for RCC metastasis presentation. Duration decreases with compromised immune response.^9^ The five‐ and 10‐year overall survival rate following pulmonary metastasectomy for metastatic renal cancer was 83% and 41.7%, respectively, according to Chen et al^14^ and 75% and 59%, respectively, according to Meacci et al.^15^

## 4 CONCLUSION

Although rare, renal cell carcinoma can present with metastases after a prolonged interval following nephrectomy. In evaluating pulmonary nodules, the possibility of metastasis from RCC should be considered in a patient even after a long time with a history of primary RCC. Even though enough data are lacking, metastasectomy is the preferred treatment for pulmonary metastases from RCC.

## CONFLICTS OF INTEREST

The authors declare that there is no potential conflict of interest with respect to the research, authorship, and/or publication of this article.

## AUTHOR CONTRIBUTIONS

ST was involved in counseling, treatment of the patient, and collection of the surgical images and CT‐scan chest images. HPD and MS examined and interpreted the pathology. MS and VA collected the required case information, images, slides, and reports and contributed to writing manuscripts. MS, VA, ASD, KN, and HPD reviewed the literature and contributed to both writing and editing the manuscript. All authors read and approved the final manuscript.

## ETHICAL APPROVAL

The hospital research board (HRB) of Nepal Cancer Hospital and Research Center, Harisiddhi, Lalitpur, Nepal provided approval.

## CONSENT

Written informed consent was obtained from the patient before the submission of the report. The signed Institutional Consent Form is on file.

## Data Availability

The data that support the findings of this study are available on request from the corresponding author.
